# When Females Produce Sperm: Genetics of *C. elegans* Hermaphrodite Reproductive Choice

**DOI:** 10.1534/g3.113.007914

**Published:** 2013-10-01

**Authors:** Adam K. Bahrami, Yun Zhang

**Affiliations:** Department of Organismic and Evolutionary Biology, Center for Brain Science, Harvard University, Cambridge, Massachusetts 02138

**Keywords:** QTL, genetic basis of, hermaphrodite reproductive strategy, natural variation, neural signaling

## Abstract

Reproductive behaviors have manifold consequences on evolutionary processes. Here, we explore mechanisms underlying female reproductive choice in the nematode *Caenorhabditis elegans*, a species in which females have evolved the ability to produce their own self-fertilizing sperm, thereby allowing these "hermaphrodites" the strategic choice to self-reproduce or outcross with males. We report that hermaphrodites of the wild-type laboratory reference strain N2 favor self-reproduction, whereas a wild isolate CB4856 (HW) favors outcrossing. To characterize underlying neural mechanisms, we show that N2 hermaphrodites deficient in mechanosensation or chemosensation (*e.g.*, *mec-3* and *osm-6* mutants) exhibit high mating frequency, implicating hermaphrodite perception of males as a requirement for low mating frequency. Within chemosensory networks, we find opposing roles for different sets of neurons that express the cyclic GMP-gated nucleotide channel, suggesting both positive and negative sensory-mediated regulation of hermaphrodite mating frequency. We also show that the ability to self-reproduce negatively regulates hermaphrodite mating. To map genetic variation, we created recombinant inbred lines and identified two QTL that explain a large portion of N2 × HW variation in hermaphrodite mating frequency. Intriguingly, we further show that ∼40 wild isolates representing *C. elegans* global diversity exhibit extensive and continuous variation in hermaphrodite reproductive outcome. Together, our findings demonstrate that *C. elegans* hermaphrodites actively regulate the choice between selfing and crossing, highlight the existence of natural variation in hermaphrodite choice, and lay the groundwork for molecular dissection of this evolutionarily important trait.

The paradox of sex has long been a problem in evolutionary biology. Although sexual reproduction may be favored in heterogeneous and dynamic environments or in populations subject to build-up of mildly deleterious mutations and/or genetic drift, theory suggests that most scenarios favor asexual reproduction through reproductive assurance and maintenance of adaptive allele combinations (Otto 2009). Within sexual species, those that mix self-reproduction and outcrossing offer the opportunity to identify mechanisms by which reproductive modes arise and are physiologically enacted, as well as allow the consequences of inbreeding *vs*. outcrossing to be quantified (Charlesworth 2006; Cutter *et al.* 2009; Morran *et al.* 2009).

The globally distributed nematode *C. elegans* comprises two sexes, hermaphrodites and males. Hermaphrodites self-reproduce through internal self-fertilization with their own sperm and can outcross with males. Phylogenetic evidence suggests that *C. elegans* evolved from a true male/female ancestor (Kiontke *et al.* 2004), and developmental evidence suggests that these hermaphrodites are somatic females that have gained the ability to produce a limited amount of self-sperm through developmental regulation of germline sex determination (Nayak *et al.* 2005; Baldi *et al.* 2009).

The life-history transition from obligate to facultative outcrossing allows *C. elegans* hermaphrodites two reproductive options, but it is not clear to what extent they make a strategic choice between them, how that choice is regulated, or whether there is genetic variation in reproductive decision segregating in natural populations. Population genetic data show that selfing is the primary mode of *C. elegans* reproduction; however, studies have identified significant rates of outcrossing in natural populations (Barriere *et al.* 2005; Sivasundar and Hey 2005), suggesting that hermaphrodite reproduction could be a naturally variable, regulated decision.

Here, we show that *C. elegans* hermaphrodite reproductive outcome (outcrossing *vs.* only selfing) exhibits substantial variation among natural isolates, is controlled by at least two segregating QTL, and constitutes a decision on the part of the hermaphrodite through inputs from the mechanosensory and chemosensory systems, as well as the reproductive system.

## Materials and Methods

### Strains

Strains were maintained at 20° under standard conditions (see Supporting Information, File S2 for strain list and details of strain construction).

### Hermaphrodite mating frequency assay

For each mating test replicate, one L4 hermaphrodite and one L4 male were transferred to a standard 20-cm^2^ NGM plate seeded with *E. coli* OP50 food and allowed to interact for 48 hr at 20°. The reproductive outcome of the interaction (*i.e.*, outcrossing or only selfing) was quantified by scoring the presence or absence of males among the F1 progeny; presence of male progeny (typically 20–100 among hundreds) was scored as "mating success," and absence of male progeny (≤2 males, but usually zero) was scored as "mating failure" (Figure S1). "Mating frequency" is plotted as the proportion of replicates with "mating success." Unless otherwise noted, HW males were used as the tester strain to determine the propensity of hermaphrodite genotypes to mate or only self (see File S2 for additional details).

### Quantitative genetic analysis

Recombinant inbred lines (RILs) were generated by intercrossing N2 and CB4856 (HW) and then selecting single hermaphrodite self-progeny for 7–10 generations from several hundred independent F2-derived lines, resulting in a panel of largely homozygous strains with mixed parental contribution from N2 and HW. One hundred fifty-nine RILs were tested in the hermaphrodite mating frequency assay, and partial genotypes of RILs were obtained by scoring a well-defined marker set (Davis *et al.* 2005) with additional markers (Wicks *et al.* 2001). QTL mapping was conducted using interval mapping (using the imputation algorithm to account for partial RIL genotypes) as implemented in rQTL (Broman *et al.* 2003) (see File S2 for additional details).

## Results

### Natural variation in hermaphrodite mating frequency

**Figure 1:**
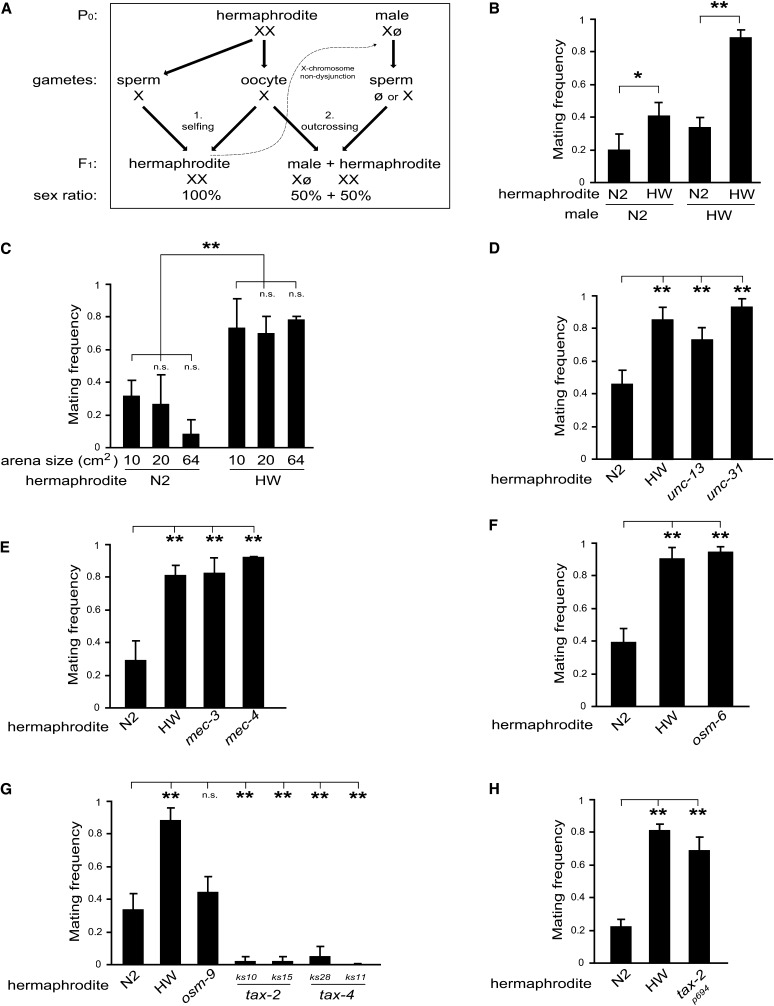
Genetic variation in *C. elegans* hermaphrodite mating frequency. (A) *C*. *elegans* life history schematic depicting alternative reproductive strategies of hermaphrodites: (1) self-reproduction *vs.* (2) outcrossing with males. Zygotes with two X chromosomes develop as hermaphrodites, whereas zygotes with only one X chromosome develop as males. Because males have only one X chromosome (ø signifies the absence on an X), 50% of their progeny are male. Males can also be generated by nondysjunction of an X chromosome during hermaphrodite meiosis (depicted by dotted line) at a low rate (∼10^−2^–10^−3^). (B) Mating frequency of single N2 and HW wild-type hermaphrodites paired with single males. Mating frequency is defined as the proportion of pairs tested bearing male F1 progeny, indicative of mating (see Supporting Information, File S2). (C) Hermaphrodite mating frequency of N2 and HW across three arena (*i.e.*, plate) sizes. The standard condition is 20 cm^2^ in all other experiments. (D–H) Hermaphrodite mating frequency of N2-derived mutants defective in (D) neurotransmission (*unc-13*) or neurosecretion (*unc-31*), (E) mechanosensation to gentle touch (*mec-3* and *mec-4*), (F) ciliated sensory neuron function (*osm-6*), (G) TRPV channel (*osm-9*) or cGMP-gated (*tax-2* and *tax-4*) channel–mediated sensory transduction in chemosensory neurons, and (H) cGMP-gated channel (*tax-2*) sensory transduction in only four sensory neurons. Unless otherwise noted, HW males were used as the tester strain to dissect hermaphrodite mating frequency. Bar graphs depict mean ± SEM of multiple trials. *P < 0.05 and **P < 0.01 by permutation test stratified by trial. n.s., not significant.

To quantify the choice between self-reproduction and outcrossing in individual hermaphrodites, we developed a simple mating frequency assay that takes advantage of the X-chromosome–based sex determination system of *C. elegans* (Hodgkin 1987). Successful cross-fertilization results in a large proportion of male progeny (approaching the theoretical maximum of 50% with complete outcrossing); in contrast, if mating is unsuccessful, then hermaphrodite self-reproduction results in nearly 100% hermaphrodite progeny, with a very low frequency of males produced by spontaneous X-chromosome nondisjunction (∼1/100 to 1/1000) (Hodgkin *et al.* 1979; Teotonio *et al.* 2006) (Figure 1A). Thus, as an assay for mating frequency, we placed one virgin L4 hermaphrodite and one virgin L4 male on standard laboratory plates (6-cm diameter with *E. coli* as food) and scored the sex of the progeny after 2 d of potential interaction (see File S2). Plates with significant male F1 progeny indicate successful mating, and we define mating frequency as the proportion of hermaphrodite/male pairs that produce male progeny (Figure S1).

Using this assay, we first asked whether two wild-type isolates of *C. elegans*—the laboratory reference strain N2, isolated in Bristol, United Kingdom, and the strain CB4856, collected in Hawaii (henceforth HW)—exhibited variation in hermaphrodite mating frequency. When N2 and HW hermaphrodites were matched with either N2 or HW males in our assay, we observed that fewer N2 hermaphrodites successfully mated compared with HW (Figure 1B), consistent with the hypothesis that hermaphrodites of different isolates favor self-reproduction or outcrossing. We also observed that HW males were more efficient at mating than N2 males (Figure 1B), confirming previous observations (Teotonio *et al.* 2006); thus, we used HW males as the tester strain in subsequent experiments (unless otherwise noted) to dissect hermaphrodite mating frequency.

To find out if the difference in mating frequency between N2 and HW hermaphrodites might represent an active process, we first manipulated the arena size of the assay. We found that the difference between N2 and HW hermaphrodite mating frequency remained at three arena sizes tested (Figure 1C). Intriguingly, N2 hermaphrodites still mated with males at a low frequency even in the smallest arena size (*i.e.*, area = 10 cm^2^). Moreover, when tested in a separate experiment on very large plates (area = 175 cm^2^), HW hermaphrodites still exhibited relatively high mating frequency (63%, N = 24 pairs) compared to N2 (9%, N = 22 pairs) (*P* < 0.001), showing that HW hermaphrodites are highly prone to mating even when the encounter rate is likely to be low.

To examine the hypothesis that N2 hermaphrodites actively resist mating with males, we quantified hermaphrodite mating frequency of N2-derived mutants defective in neural function. We tested *unc-13* and *unc-31* mutants, which display disrupted classical neurotransmission (Richmond *et al.* 1999) or dense-core vesicle-mediated neurosecretion (Avery *et al.* 1993), respectively, and which are largely paralyzed. We observed that both *unc-13* and *unc-31* mutant hermaphrodites exhibited high mating frequency, similar to HW (Figure 1D). This result confirms that N2 hermaphrodites are physiologically able to mate, and the low mating phenotype of N2 is not attributable to postzygotic incompatibility or selection against cross-progeny. Together, these data demonstrate natural variation in hermaphrodite mating frequency between N2 and HW and show that broad disruption of nervous system function results in increased mating in N2 hermaphrodites, revealing a behavioral program that regulates hermaphrodite mating frequency.

### Sensory contribution to hermaphrodite mating frequency

Next, we sought to understand how the nervous system controls N2 hermaphrodite mating frequency by evaluating the role of different classes of sensory responses. Because hermaphrodites and males physically interact during courtship and mating, we first investigated the requirement of a set of mechanosensory neurons to prevent mating in N2 hermaphrodites. We tested hermaphrodites carrying mutations in *mec-3* or *mec-4*, which encode a LIM-homeodomain–containing transcription factor required for cell-fate determination of the mechanosensory neurons (Way and Chalfie 1988) or a subunit of a DEG/ENaC channel necessary for transduction of mechanical stimuli (Driscoll and Chalfie 1991), respectively. Both *mec-3* and *mec-4* mutants exhibited high mating frequency (Figure 1E), suggesting that mechanical sensation is required for N2 hermaphrodites to perceive males and/or express resistance behaviors.

Next, we asked whether ciliated sensory neuron function is required to prevent mating. We examined an *osm-6* mutant, which has altered morphology of ciliated sensory neurons resulting in reduced perception of a variety of stimuli, including olfactory and gustatory cues (Collet *et al.* 1998). We observed that *osm-6* mutant hermaphrodites exhibited high mating frequency, similar to HW (Figure 1F). Because *osm-6* mutants exhibit normal locomotion in the presence of food (Gray *et al.* 2005), unlike *unc-13* and *unc-31* mutants described, it is unlikely that the high mating frequency of *osm-6* mutants results from locomotion defects. Rather, we propose that at least a subset of ciliated sensory neurons is required for N2 to resist mating with males.

To dissect sensory regulation of N2 hermaphrodite mating, we next tested two classes of mutants, *osm-9* and *tax-2/tax-4*. For sensory transduction, most *C. elegans* chemosensory neurons use either a TRPV channel encoded by *osm-9* (Colbert *et al.* 1997) or a cGMP-gated channel encoded by *tax-2* and *tax-4* (Coburn and Bargmann 1996; Komatsu *et al.* 1996). We found that *osm-9* mutant hermaphrodites exhibited mating frequency comparable to N2 (Figure 1G), suggesting that *osm-9*–expressing neurons do not play a major role in generating low mating in N2 hermaphrodites. Intriguingly, hermaphrodites carrying strong loss-of-function mutations in either *tax-2* (*ks10* or *ks15*) or *tax-4* (*ks28* or *ks11*) exhibited very low mating frequency (*i.e.*, even lower than N2) (Figure 1G). To confirm this, we rescued the *tax-4*(*ks28*) mutant with a N2 wild-type *tax-4* transgene (Figure S2). These results indicate that some *tax-2/tax-4*–expressing neurons normally promote hermaphrodite mating.

The opposite effects of loss-of-function of *osm-6* or *tax-2/tax-4* raised the possibility that different sets of sensory neurons may play opposing roles in regulating hermaphrodite mating. Intriguingly, we found that a more subtle mutation in *tax-2*, *p694*—which causes loss of TAX-2 expression (and associated chemosensory responses) in just four pairs of amphid neurons because of a deletion in the *tax-2* promoter, first exon, and first intron (Coburn and Bargmann 1996)—exhibited high mating frequency (Figure 1H), a phenotype opposite of the strong loss-of-function *tax-2* and *tax-4* mutants described. Thus, we conclude that within the TAX-expressing sensory neurons, there are at least two opposing sensory circuits regulating hermaphrodite mating frequency.

Together, these results demonstrate that neural responses through multiple sensory modalities, including chemosensory and mechanosensory, regulate N2 hermaphrodite reproductive outcome. Further, our observation that most of the mutations tested—which disrupt, rather than enhance, neural processes—resulted in increased hermaphrodite mating shows that low mating in N2 hermaphrodites constitutes a decision.

### Role of self-reproduction in hermaphrodite mating frequency

To evaluate the hypothesis that *C. elegans* hermaphrodites express low mating frequency because they have the ability to self-reproduce without males, we depleted hermaphrodite self-sperm and measured mating frequency. *C. elegans* hermaphrodites are somatically female and their germline develops sequentially, first as male producing ∼300 sperm, before irreversibly switching to egg production as female; hermaphrodite self-sperm production is finite and limits the number of progeny generated by self-reproduction (Ward and Carel 1979).

First, we measured the correlation of natural sperm depletion and mating frequency in N2 and HW hermaphrodites. As a treatment, synchronized L4 hermaphrodite larvae were allowed to self-reproduce in the absence of males for 0, 1, 2, or 3 d before being placed in the mating frequency assay. In parallel, animals from the same treatment were placed singly on plates without males to quantify the onset of sperm depletion, which is indicated when hermaphrodites begin to lay unfertilized, unviable haploid oocytes because of lack of sperm (Ward and Carel 1979).

**Figure 2:**
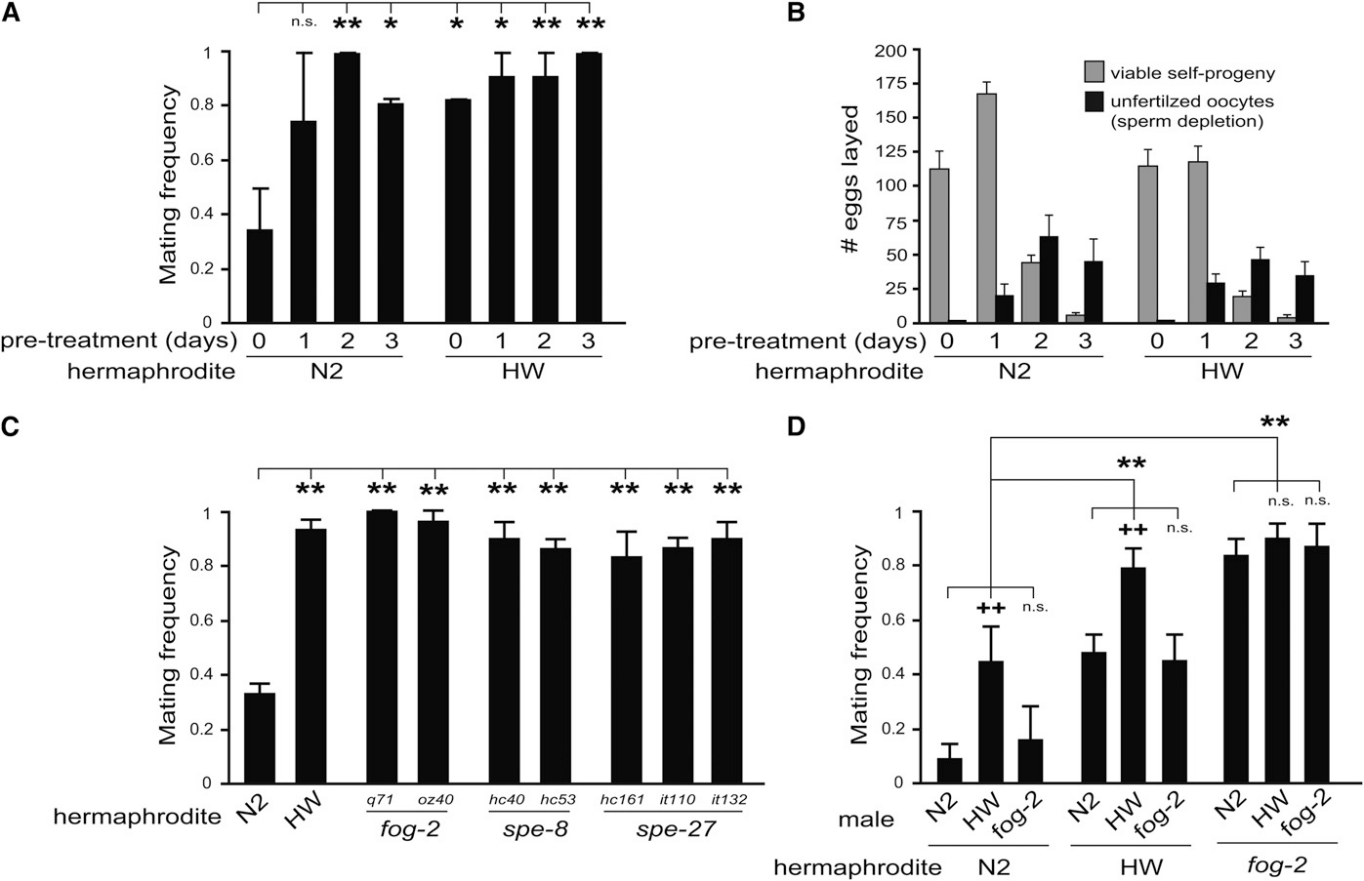
Hermaphrodite self-reproduction antagonizes mating. (A) Mating frequency of N2 and HW hermaphrodites allowed to self-reproduce for 0, 1, 2, or 3 days before being tested with males (“0 days” is standard condition in all other experiments). (B) Reproductive tempo (*i.e.*, self-progeny and self-sperm depletion) of isolated hermaphrodites in the period after the same treatment in (A). (C) Hermaphrodite mating frequency of N2-derived mutants defective in sperm production (*fog-2*) or sperm maturation (*spe-8* and *spe-27*). (D) Mating frequency of all paired combinations of N2, HW, and *fog-2*(*q71*) hermaphrodites and males. Unless otherwise noted, HW males were used as the tester strain to dissect hermaphrodite mating frequency. Bar graphs depict mean ± SEM of multiple trials. *P < 0.05 and **^++^***P* < 0.01 by permutation test stratified by trial. n.s., not significant. **Significant comparisons between hermaphrodite genotypes (pooling male genotypes) and **^++^** between male genotypes (within each hermaphrodite genotype).

We observed that, whereas N2 hermaphrodites exhibited low mating during the day 0 condition (*i.e.*, standard condition), their mating frequency increased with time (Figure 2A), in correlation with the appearance of sperm depletion (Figure 2B). This result is analogous to that found by Kleeman and Basolo (2007), showing that mating behavior is positively correlated with self-sperm depletion.

Second, to causally link self-reproduction to hermaphrodite reproductive choice, we tested two classes of N2-derived mutants disrupted in hermaphrodite self-sperm development. *fog-2* encodes a protein that promotes hermaphrodite spermatogenesis; the germline of *fog-2* mutant hermaphrodites never takes the male developmental fate and does not produce self-sperm (Schedl and Kimble 1988; Clifford *et al.* 2000). In contrast, *spe-8* and *spe-27* encode factors that promote sperm maturation; although *spe-8* and *spe-27* mutant hermaphrodites produce self-sperm, the activation step that allows sperm motility is blocked, resulting in sperm unable to fertilize eggs (L’Hernault *et al.* 1988; Minniti *et al.* 1996). We found that *fog-2*, *spe-8*, and *spe-27* mutant hermaphrodites all exhibited high mating frequency (Figure 2C). Consistent with the finding that *fog-2* expression in hermaphrodites occurs primarily during self-sperm development as larvae and is not required for spermatogenesis in males (Clifford *et al.* 2000), we demonstrated that disruption of *fog-2* only affects hermaphrodite, and not male, mating frequency (Figure 2D). Of note, Morsci *et al.* (2011) recently showed that male sexual drive in *C. elegans* depends on hermaphrodite self-reproductive status, because sensitized mutant males expressed more vigorous mating attempts with *fog-2* mutant compared to wild-type hermaphrodites. These previous studies showed that hermaphrodite self-reproduction affects male (Morsci *et al.* 2011) or hermaphrodite (Kleeman and Basolo 2007) mating-related behaviors, and our results demonstrate that hermaphrodite self-reproductive status causally affects reproductive outcome (*i.e.*, outcrossing *vs.* self-reproduction).

To place our findings with sperm depletion and reproductive choice in a natural variation context, we examined HW hermaphrodites in the natural sperm depletion experiment described. Because HW hermaphrodites begin adulthood more permissive to mating and remain so (Figure 2A), we hypothesized that they might produce fewer self-sperm or deplete their sperm earlier. Contrary to this prediction, we observed that HW had a largely similar tempo of self-reproduction compared to N2 (Figure 2B), and there was no obvious correlation with the appearance of self-sperm depletion and mating frequency (*e.g.*, compare N2 *vs.* HW during day 0 and day 1 treatments in Figure 2, A and B). This finding suggests variation among isolates regarding how self-reproductive cues are used to regulate mating *vs.* selfing.

### Genetic architecture underlying hermaphrodite mating frequency

**Figure 3:**
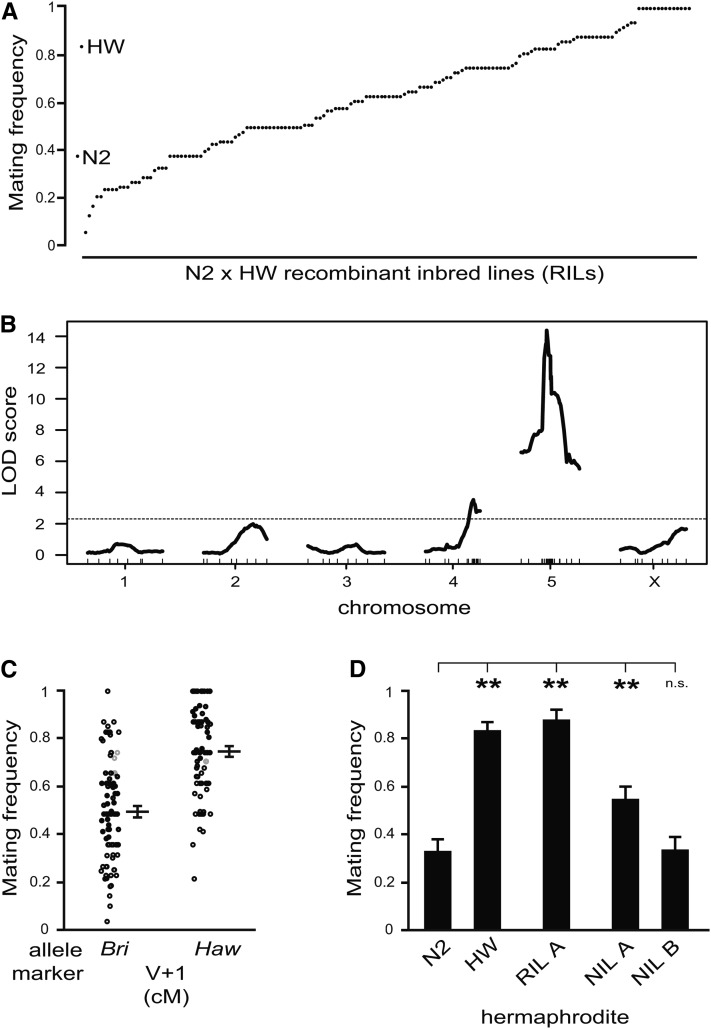
QTL mapping of hermaphrodite mating frequency variation between N2 and HW. (A) Hermaphrodite mating frequency of 158 recombinant inbred lines generated from a cross between N2 and HW (see Supporting Information, File S2). (B) QTL analysis of hermaphrodite mating frequency of RILs in (A). Dotted line depicts significance threshold determined by permutation of the data. (C) Effect size of *mate-1* QTL on chromosome V at a linked marker (V + 1 cM). Black points denote phenotypes of RILs with genotype data at this marker; gray points denote phenotypes of remaining RILs with imputed genotypes at this marker. “*Bri*” indicates N2 allele; “*Haw*” indicates HW allele. Average values are mean ± SEM. (D) Hermaphrodite mating frequency of near-isogenic lines (NIL A and NIL B) generated from RIL A carrying ∼5-MB or ∼3-MB HW haplotype around *mate-1*, respectively. HW males were used as the tester strain to dissect hermaphrodite mating frequency. Bar graphs depict mean ± SEM of multiple trials. **P < 0.01 by permutation test stratified by trial. n.s., not significant.

To find out what genetic differences underlie natural variation in hermaphrodite mating frequency, we generated a panel of RILs by interbreeding the N2 and HW wild-type isolates and mapped mating frequency to SNP markers spread across the *C. elegans* genome (see File S2). Hermaphrodite mating frequency segregated among 158 RILs (Figure 3A), and we uncovered two QTL explaining a significant proportion of the phenotypic variation, one major QTL in the middle of chromosome V and one weaker QTL on the right side of chromosome IV, which we named *mate-1* and *mate-2*, respectively (Figure 3B).

As a control, we also scored and mapped aggregation behavior, a trait known to vary between N2 and HW. As expected, we identified a major QTL centered on *npr-1* (Figure S3), which encodes a G-protein-coupled receptor with a well-described polymorphism affecting various *C. elegans* behaviors, including aggregation (de Bono and Bargmann 1998). Importantly, our mapping experiments for mating frequency did not detect any QTL near *npr-1*, but found strong evidence for association elsewhere in the genome. Further, we found that the N2-derived *npr-1(ad609)* mutant—which displays aggregation behavior similar to HW—had wild-type N2 hermaphrodite mating frequency (Figure S4).

We chose to further characterize the *mate-1* QTL. Consistent with the possibility that multiple loci affect mating frequency variation between N2 and HW, the average effect size of alternate alleles at a marker near the *mate-1* QTL peak in our RIL population was ∼0.25 (on a scale of 0 to 1) (Figure 3C), which is smaller than the mating frequency difference between the parent strains (∼0.45) (Figure 3A).

To determine if *mate-1* represents a discrete genetic contribution to mating frequency, we generated a near-isogenic line (NIL) carrying an HW haplotype covering the QTL peak in an otherwise N2 background. We observed that NIL A exhibited mating frequency significantly higher than N2 (Figure 3D), confirming that *mate-1*(*HW*) is sufficient to increase hermaphrodite mating. The increase in mating frequency of NIL A relative to N2 is similar to the mean effect size estimated from the RIL population (Figure 3C), but it cannot fully explain the difference between N2 and HW or RIL A. Further, although RIL A displayed mating frequency comparable to that of HW (Figure 3D), it carries a N2 haplotype across the *mate-2* locus on chromosome IV. Together, these results suggest that additional, unidentified loci contribute to N2/HW mating variation.

In the context of generating NIL A, which contains the entire ∼5-MB HW fragment covering *mate-1* from RIL A, we also obtained a recombinant that lost the left half of the HW fragment in the region, leaving a ∼3-MB introgression. This NIL B exhibited low mating frequency, similar to N2 (Figure 3D), suggesting that the right half of the fragment is not sufficient to augment mating. This implies that the left half of the fragment could be sufficient alone, or that both are required.

### Genetic diversity of *C. elegans* hermaphrodite mating frequency

**Figure 4:**
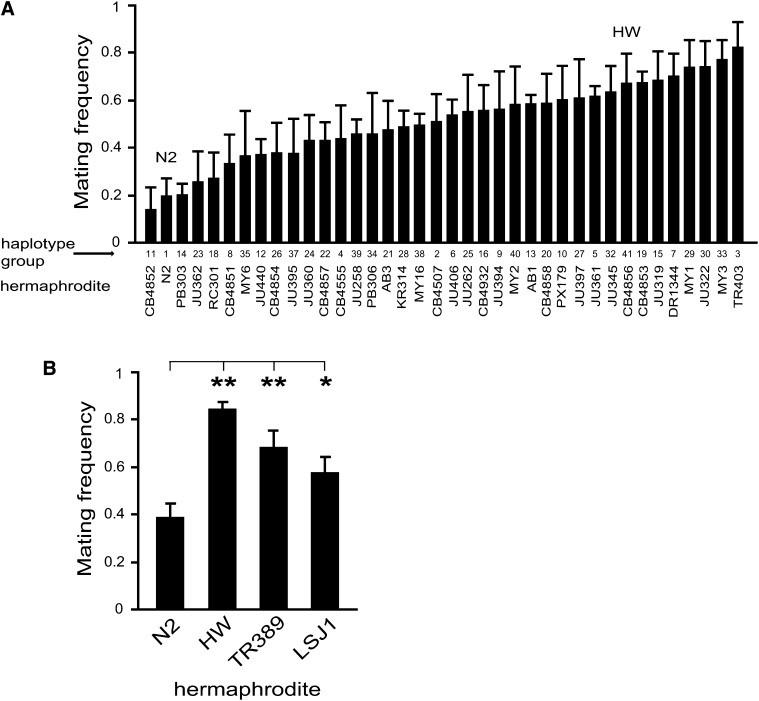
Hermaphrodite mating frequency variation among diverse wild-type isolates. (A) Hermaphrodite mating frequency of 38 wild-type isolates representing haplotype diversity of *C. elegans*. (B) Hermaphrodite mating frequency of two additional haplotype group “1” isolates, TR389 and LSJ1, that are genetically similar to N2. Haplotypes defined by Rockman and Kruglyak (2009) and McGrath *et al.* (2009). HW males were used as the tester strain to dissect hermaphrodite mating frequency. Bar graphs depict mean ± SEM of multiple trials. *P < 0.05 and **P < 0.01 by permutation test stratified by trial.

Next, to find out if N2 *vs.* HW variation is representative of *C. elegans* as a whole, we extended our analysis to include wild-type isolates representing 38 of 41 unique haplotype groups known to encompass most of the *C. elegans* global diversity (Rockman and Kruglyak 2009). Among these strains, we observed a continuum of hermaphrodite mating frequency from low to high, with N2 among those favoring selfing and HW among those favoring outcrossing (Figure 4A). With the caveat that these strains were collected over several decades and maintained in laboratory environments, this pattern of behavioral variation suggests that hermaphrodite mating frequency likely varies in natural populations. Although it is not clear if this pattern reflects past neutral or selective forces, because genetic variation is a prerequisite for natural selection to act, these data raise the possibility that alternative reproductive strategies compete in natural populations.

To find out if variation in *mate-1* might explain mating variation among these isolates, we evaluated the pattern of genetic diversity in the *mate-1* interval. Strikingly, in comparison with most of the genome, Rockman and Kruglyak (2009) identified near-complete allelic homogeneity in the middle of chromosome V among the 41 haplotypes, possibly because of a selective sweep and associated hitchhiking of neighboring loci. Although almost all tested isolates carry a Bristol (*i.e.*, N2) allele across the *mate-1* region, yet we observed continuous variation in hermaphrodite mating frequency (Figure 4A), we conclude that the *mate-1* QTL identified between N2 and HW cannot solely explain the pattern of phenotypic diversity observed across this panel of isolates.

Striking in our natural isolate data is the observation that strains carrying very similar haplotypes exhibited the full range of mating frequency (Figure 4A). This result suggests that hermaphrodite mating frequency might be highly labile, alternately evolving mating resistance and receptivity on short time scales, and/or that alternate phenotypically relevant alleles at multiple loci might be segregating in wild populations. Using polymorphism data and association mapping methods (Rockman and Kruglyak 2009), we failed to identify any significant association between genotype and hermaphrodite mating frequency for the 38 wild isolates tested here (data not shown). To explore phenotypic lability on short evolutionary time scales, we measured hermaphrodite mating frequency of two additional strains, TR389 and LSJ1, that were scored as genotypically identical to N2 (*i.e.*, haplotype group “1”) using a panel of 1460 SNPs (Rockman and Kruglyak 2009; McGrath *et al.* 2009). We found that both TR389 and LSJ1 exhibited high hermaphrodite mating frequency (Figure 4B). Subsequent analysis has shown that these strains harbor a variety of sequence differences relative to N2 as a result of possible laboratory evolution (McGrath *et al.* 2009; Weber *et al.* 2010; McGrath *et al.* 2011), and our results show that hermaphrodite mating frequency can be a labile trait, possibly because of a large mutational target of genes that regulate reproductive decision.

## Discussion

### It takes two to tango

*C. elegans*, as a species, appears to be far along the path toward complete self-reproduction, as evidenced by a suite of traits related to the degeneration of outcrossing, termed the “selfing syndrome,” such as reduced fitness of hybrid genotypes, reduced pheromonal attraction from hermaphrodites, and diminished male function in mating tests (Garcia *et al.* 2007; Chasnov *et al.* 2007; Cutter 2008). However, males have not gone extinct in this species, implicating the periodic or context-dependent importance of outcrossing in the field. Our results highlight the coexistence of self-reproduction and outcrossing in *C. elegans* as a strategic game and identify hermaphrodite behavior as an important axis of variation regulating this trade-off.

How do *C. elegans* hermaphrodites regulate whether they mate with males or self-reproduce? We provide evidence for a multifaceted role of the sensory system in regulating this decision. Because mechanosensory and ciliated sensory (*osm-6*) neurons are required for hermaphrodites to resist mating (Figure 1, E and F), we propose that hermaphrodites perceive physical and/or pheromonal cues from the male that trigger the expression of resistance behavior. Intriguingly, we found opposite effects on mating frequency for two sets of sensory neurons expressing TAX channels (Figure 1, G and H). This result demonstrates that the *C. elegans* nervous system is capable of both up-regulation and down-regulation of hermaphrodite mating frequency, a prerequisite for the expression of a decision. The cellular basis of these competing signaling interactions, or whether they represent one or more behavioral outputs, remains to be investigated further.

It is largely unknown how behaviors evolve after a major shift in life history. Recent work (Nayak *et al.* 2005; Baldi *et al.* 2009) has implicated self-sperm specification and activation as two traits responsible for the origin of hermaphroditism in *C. elegans*. Specifically, *fog-2* appears to be a recently derived *C. elegans* lineage-specific gene required for hermaphrodites to produce sperm (Nayak *et al.* 2005). In addition, sperm maturation in *C. elegans* hermaphrodites requires *spe-8* and *spe-27* signal transduction (L’Hernault *et al.* 1988; Minniti *et al.* 1996). By comparison, *C. elegans* males do not require *fog-2* to produce sperm and use two redundant signaling pathways (involving *spe-8/spe-27* or *try-5*) to activate sperm (Smith and Stanfield 2011). The gain of self-reproduction through self-sperm production is expected to select for reduction in the mating drive of these newly evolved hermaphrodites. Consistently, we found that N2 hermaphrodites naturally depleted of self-sperm (Figure 2A) or carrying mutations that disrupt germline sperm specification or sperm activation (Figure 2C) expressed increased mating. Thus, we speculate that an additional step in the evolution of hermaphroditism is to reinforce self-reproduction through behavioral expression of reduced mating. Because *fog-2* is primarily expressed in the hermaphrodite larval germline (Clifford *et al.* 2000), it is unlikely that *fog-2* mediates behavior directly. Rather, we speculate that an internal cue, representing self-reproductive status in mature adults, informs mating behavior generated by the nervous system.

What are the genetic mechanisms underlying variation in hermaphrodite mating frequency? We identified two QTL, *mate-1* and *mate-2*, that account for a large portion of the variation between two strains, N2 and HW (Figure 3B). However, it is unlikely that the *mate-1* locus is solely responsible for the observed continuous, quantitative phenotypic variation in the mating frequency among other wild-type isolates (Figure 4A), because the middle of chromosome V that spans the *mate-1* locus exhibits almost no variation among these strains and nearly all strains carry an N2 allele (Rockman and Kruglyak 2009). This result indicates that additional genes are likely responsible for variation in mating frequency.

We speculate that behaviors favoring mating are more likely to be the ancestral reproductive state of *C. elegans*, based on our results with sperm mutants that developmentally phenocopy the hypothesized female ancestral state of *C. elegans*. Further, the observation that females of *C. remanei*, a closely related obligate outcrossing species, are much more attractive to heterospecific *C. elegans* males than are conspecific *C. elegans* hermaphrodites (Chasnov *et al.* 2007) suggests that early *C. elegans* hermaphrodites may have been more attractive or willing to mate with males than they are today.

## Supplementary Material

Supporting Information
