# Supplementary material for: When Females Produce Sperm: Genetics of C. elegans Hermaphrodite Reproductive Choice
Source: G3 (Bethesda). 2013 Oct 1;3(10):1851–9. doi: 10.1534/g3.113.007914 (PMC3789810; doi:10.1534/g3.113.007914)
Supplement: Supporting Information [file supp_3_10_1851__index.html]

When Females Produce Sperm: Genetics of C. elegans Hermaphrodite Reproductive Choice — Supporting Information 

# When Females Produce Sperm: Genetics of *C. elegans* Hermaphrodite Reproductive Choice

## Supporting Information for Bahrami and Zhang, 2013

**Files in this Data Supplement:**

- Supporting Information - Figures S1-S6 and Files S1-S2 (PDF, 1 MB)
- Figure S1 - Quantification of hermaphrodite and male progeny in mating frequency assay (PDF, 264 KB)
- Figure S2 - Transgenic rescue of the *tax-2* (*ks28*) mutant recapitulates wild-type N2 hermaphrodite mating frequency. (PDF, 274 KB)
- Figure S3 - QTL mapping of aggregation behavior and hermaphrodite mating frequency variation between N2 and HW (PDF, 283 KB)
- Figure S4 - The *npr-1* (*ad609*) mutant does not exhibit increased hermaphrodite mating frequency. (PDF, 277 KB)
- Figure S5 - Relationship between rank percentile measurements of mating frequency of RILs within subgroups tested and the combined dataset (PDF, 288 KB)
- Figure S6 - Transgenic line experiments do not implicate *glb-5* as the causal gene underlying the *mate-1* QTL. (PDF, 284 KB)
- File S2 - SI Materials and Methods (PDF, 450 KB)
- File S1 - Complete set of raw data (.xls, 300 KB)
