# Supplementary material for: When Females Produce Sperm: Genetics of C. elegans Hermaphrodite Reproductive Choice
Source: G3 (Bethesda). 2013 Oct 1;3(10):1851–9. doi: 10.1534/g3.113.007914 (PMC3789810; doi:10.1534/g3.113.007914)
Supplement: Supporting Information [file supp_g3.113.007914_007914SI.pdf]

G3 Genes | Genomes | Genetics

When Females Produce Sperm: Genetics of *Caenorhabditis elegans* Hermaphrodite Reproductive Choice

Adam K. Bahrami & Yun Zhang*
Department of Organismic & Evolutionary Biology, Center for Brain Science, Harvard University, 52 Oxford St, Cambridge, MA 02138

*corresponding author: yzhang@oeb.harvard.edu

DOI: 10.1534/g3.113.007914

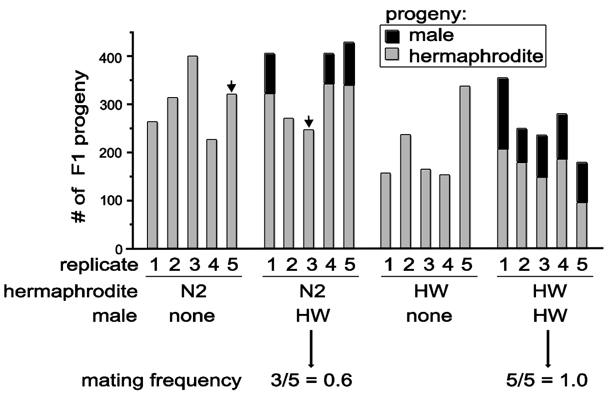

Figure S1. Quantification of hermaphrodite and male progeny in mating frequency assay
A control experiment depicting the outcome of alternative hermaphrodite reproductive strategies. In each replicate, one L4 hermaphrodite is placed alone or paired with one L4 male in an arena. During the test period, the hermaphrodite may mate with the male, resulting in a large number of male progeny ('mating success'), or only self-reproduce, resulting in nearly 100% hermaphrodite progeny ('mating failure'). Mating frequency is defined as the proportion of tested pairs bearing large numbers of male F1 progeny. Hermaphrodites tested alone, where self-reproduction is the only option, produce zero or, occasionally, very few males due to X-chromosome nondysjunction. Arrowheads depict the two replicates where a single F1 male was observed among progeny, showing that the effective male progeny rate from nondysjunction is orders of magnitude less than that from mating in our assay.

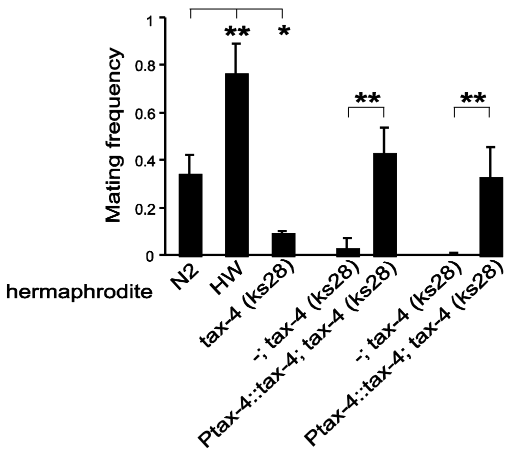

Figure S2. Transgenic rescue of the *tax-4 (ks28)* mutant recapitulates wild-type N2 hermaphrodite mating frequency

Mating frequency of two transgenic strains, ZC2302 and ZC2303 [and their nontransgenic siblings, depicted by a 'minus' sign], expressing a full-length N2 *tax-4* genomic DNA fragment in a *tax-4 (ks28)* background. Bar graphs depict mean±SEM of multiple trials. *$p<0.05$ and **$p<0.01$ by permutation test stratified by trial.

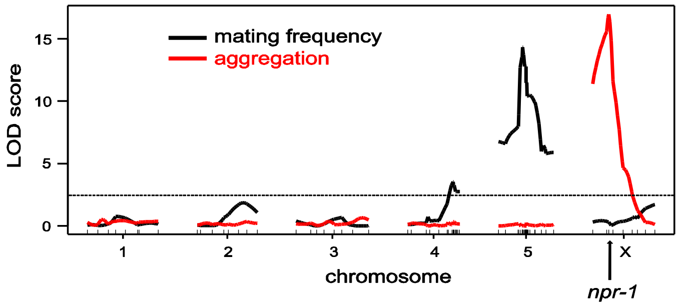

Figure S3. QTL mapping of aggregation behavior and hermaphrodite mating frequency variation between N2 and HW

As a control, variation in aggregation behavior between N2 and HW was mapped to a marker adjacent to *npr-1*. Using the same RILs, mating frequency mapped to two QTL, *mate-1* and *mate-2*, on chromosomes V and IV, respectively, but did not map to *npr-1*. In this analysis, aggregation was scored and mapped as a binary trait. Dotted line depicts significance threshold determined by permutation of the data.

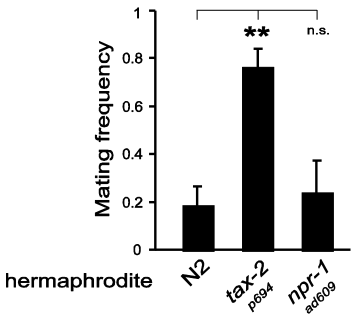

Figure S4. The *npr-1 (ad609)* mutant does not exhibit increased hermaphrodite mating frequency

The *npr-1 (ad609)* mutant, which displays bordering and aggregation behaviors, does not exhibit increased hermaphrodite mating frequency relative to N2, demonstrating that bordering and aggregation are not sufficient to cause high mating. The *tax-2 (p694)* mutant is included here as a high mating control. Bar graphs depict mean±SEM of multiple trials. **$p<0.01$ by permutation test stratified by trial; n.s., not-significant.

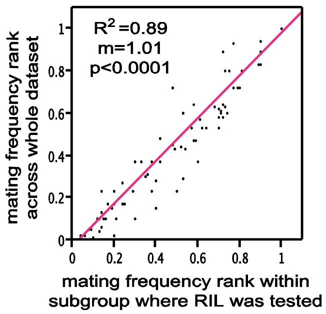

Figure S5. Relationship between rank percentile measurements of mating frequency of RILs within subgroups tested and the combined dataset

Mating frequency of 158 RILs was measured in subgroups of 22-25 RILs. Mating frequency data were used to calculate rank assignments for each RIL within subgroup tested (X-axis) and across the 158 RIL combined dataset as a whole (Y-axis). The strong linear correlation observed suggests that data collected as subgroups could be combined for QTL analysis.

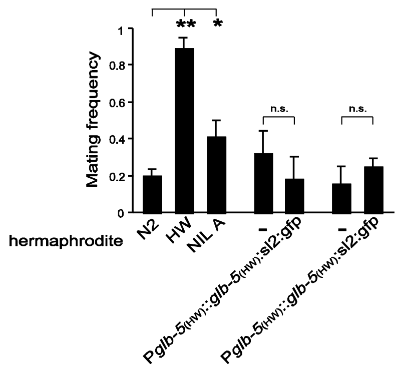

Figure S6. Transgenic line experiments do not implicate *glb-5* as the causal gene underlying the *mate-1* QTL

Mating frequency of two transgenic strains, ZC1902 and ZC1903 [and their nontransgenic siblings, depicted by a 'minus' sign], carrying ectopic expression of *glb-5(HW)* in an N2 background. These transgenic constructs conferred *glb-5*-dependent phenotypes in a previous study (McGrath *et al*. 2009), but had no effect on mating frequency here. Bar graphs depict mean±SEM of multiple trials. *p<0.05 and **p<0.01 by permutation test stratified by trial; n.s., not significant.

File S1

Complete set of raw data

Available for download at http://www.g3journal.org/lookup/suppl/doi:10.1534/g3.113.007914/-/DC1

File S2

SI Materials and Methods

Reference, mutant, and transgenic strains

N2
MT7929 *unc-13(e51)*I
CB928 *unc-31(e928)*IV
CB1338 *mec-3(e1338)*IV
TU253 *mec-4(u253)*X
PR811 *osm-6(p811)*V
CX10 *osm-9(ky10)IV*
FK100 *tax-2(ks10)*I
FK101 *tax-2(ks15)*I
PR694 *tax-2(p694)*I
FK103 *tax-4(ks28)*III
FK129 *tax-4(ks11)*III
CB4108 *fog-2(q71)*V
BS553 *fog-2(oz40)*V
BA785 *spe-8(hc40)*I
BA786 *spe-8(hc53)*I
BA947 *spe-27(hc161*)IV
BA900 *spe-27(it110)*IV
BA953 *spe-27(it132)*IV
DA609 *npr-1(ad609)*X
ZC2302 *tax-4(ks28)*III; yxEx1204 [*Ptax-4::tax-4, Punc-122::rfp*]
ZC2303 *tax-4(ks28)*III; yxEx1205 [*Ptax-4::tax-4, Punc-122::rfp*]
ZC1902 kyEx2802 [*Pglb-5(HW)::glb-5(HW):sl2:gfp; Pelt-2::mCherry*]
ZC1903 kyEx2803 [*Pglb-5(HW)::glb-5(HW):sl2:gfp; Pelt-2::mCherry*]

wild-type (natural) isolates

AB1, AB3, CB4507, CB4555, CB4851, CB4852, CB4853, CB4854, CB4856 (HW), CB4857, CB4858, CB4932, DR1344, JU258, JU262, JU319, JU322, JU345, JU360, JU361, JU362, JU394, JU395, JU397, JU406, JU440, KR314, LSJ1, MY1, MY16, MY2, MY3, MY6, N2, PB303, PB306, PX179, RC301, TR389, TR403

Recombinant inbred and near-isogenic lines

ZC428-ZC588 [excluding ZC453, ZC456 and ZC572] and ZC870: 159 N2 x HW recombinant inbred lines (RILs) (see **File S1**)
ZC1605 yxIR1 (V, CB4856>N2)V: near isogenic line "NIL A" around *mate-1*
ZC1901 yxIR2 (V, CB4856>N2)V: near isogenic line "NIL B" around *mate-1*

Mating frequency assay

Mating test arenas were prepared by seeding standard 20cm^2 NGM plates 18hr prior to use with 50uL of a saturated OP50 *E. coli* culture (bacterial lawn area = ~16cm^2). *C. elegans* strains were prepared by first allowing rapidly growing populations to starve for 1-3 days, resulting in enrichment of growth-arrested L1 larvae. 48hr after transfer to new food, a stage-synchronized pair of one L4 hermaphrodite and one L4 male were transferred to each mating test arena and incubated at 20° for 48hr (*i.e.* the mating test). L4 animals develop sexual maturity during the first several hours; subsequent interaction over the course of the assay may result in mating behavior that leads to fertilization and the generation of cross-progeny and/or result in self-reproduction of the hermaphrodite alone. After the 48hr test period, both male and hermaphrodite were removed and eggs laid during the test were left to hatch and develop. After an additional 48hr, sex of the progeny was scored visually; presence of substantial male progeny

(typically 20-100 of hundreds) was scored as a 'mating success' and absence of male progeny (≤ 2 males, but usually zero) was scored as a 'mating failure' (**Figure 1A** and **Figure S1**). Mating frequency is plotted as the proportion of replicate pairs resulting in 'mating success,' and, thus represents the reproductive 'decision' of each hermaphrodite. Each experiment comprised multiple trials with typically 5-10 replicate pairs per treatment in each trial (see **File S1** for raw data). Unless otherwise noted, HW males were used as the tester strain to dissect hermaphrodite mating frequency. Statistical significance between pairs of treatments was determined by employing a randomization test, stratified by trial. In the stratified randomization test, data were permuted within trial before summing the test statistics across trials to increase power and reduces false positive rate when sample sizes vary among trials. In practice, we found that the standard permutation test (without stratification) gave nearly identical results compared to including stratification by trial. Throughout, 10,000 permutations of the data in each comparison were used to generate the distribution of test statistics that made up the null distribution.

Sperm depletion analysis

Following Kleeman and Basolo (2007), we quantified viable progeny by counting hatched larvae and unhatched, oblong eggs containing a visible embryo, and quantified sperm depletion by counting circular, undeveloped eggs that had been laid without a defined embryo, representing unfertilized, haploid oocytes (Ward and Carell 1979). We differ from Kleeman and Basolo's approach in the manner of our treatment condition. Whereas they correlated sperm depletion and mating resistance in unmanipulated hermaphrodites, we created cohorts of individuals that were allowed to self-reproduce for 0, 1, 2 or 3 days on NGM plates seeded with OP50 *E. coli* food before measuring their mating frequency and self-reproductive potential.

Hermaphrodites of the mutants *fog-2*, *spe-8* and *spe-27* are self-sterile, due to disruption of self-sperm specification or activation (Schedl and Kimble 1988, L'Hernault *et al*. 1988, Minniti *et al*. 1996, Clifford *et al*. 2000). However, they produce viable cross progeny after mating with males. Further, *spe-8* and *spe-27* mutant hermaphrodites also produce self-progeny after mating with males, because male seminal fluid can *trans*-activate hermaphrodite sperm. Thus, in our mating assays with *fog-2*, *spe-8* and *spe-27* mutants, we scored the absence of progeny as 'mating failure' and the presence of progeny, which always included male progeny, as 'mating success.'

Generation of RIL population, phenotyping, genotyping and QTL mapping

To generate a RIL population for mapping, we conducted reciprocal crosses between N2 and HW. Hermaphrodite F1 cross-progeny (identified retroactively by genotyping a SNP marker) were allowed to self. Several hundred F2 hermaphrodites were used to initiate selfing lines that were then singled for 7-10 generations to generate a panel of unique, largely homozygous lines with mixed genomic contributions from N2 and HW.

Measuring hermaphrodite mating frequency of 159 RILs was facilitated by dividing and testing them in seven groups with 22-25 RILs each, plus N2 and HW controls. Rank mating frequency measured for each RIL within its test group strongly correlated with rank calculated across the whole dataset (**Figure S5**), allowing us to combine the phenotype data for QTL analysis. In parallel, as a control for mapping with our RILs, we scored a binary trait, aggregation of hermaphrodites at the border of the *E. coli* lawn (de Bono and Bargmann 1998).

We genotyped select RILs at 48 SNP markers (8/chromosome), using a well-defined PCR-based snip-SNP protocol (Davis *et al.* 2005). Due to our partial genotyping strategy, we used published genetic map information,

rather than the map inferred from our cross. Once we identified the *mate-1* and *mate-2* QTL, we added additional markers (Wicks *et al.* 2001) [accessed at genome.wustl.edu/services/c-elegans-snpdata] in the relevant intervals to narrow locations of recombination breakpoints for informative RILs, resulting in a total of 85 markers. PCR results were robust and restriction digest patterns were as expected. For every marker, we confirmed that N2 and HW gave the expected allelic digest pattern. Our phenotype and genotype data are found in **File S1**. We mapped mating frequency and aggregation behavior in our RILs using standard interval mapping methods implemented in rQTL (Broman and Sen 2009). Single QTL scans for mating frequency using the imputation algorithm [to account for partial genotyping of our RILs] identified *mate-1* and *mate-2*; further analysis using epistasis and stepwise model selection algorithms did not identify additional QTL or interactions.

Transgenic rescue of the *tax-4 (ks28)* with a N2 wild-type *tax-4* transgene

To generate a *tax-4* rescue transgene, a genomic DNA fragment including the native *tax-4* promoter, coding sequence, and 3'UTR was amplified from N2 genomic DNA by PCR using the primers:

tax-4(g)_F1 5' GCGTGCAAAAGCCGTAAAAGATG 3'
tax-4(g)_R1 5' AGCATCACACTTTCCAGACCAATC 3'

The PCR product was purified and injected at 8ng/uL— along with *Punc-122::rfp* at 20ng/uL as a transformation marker— into FK103 *tax-2 (ks28)*. Two resulting stable lines expressing the transgene as extra-chromosomal arrays (ZC2302 and ZC2303) were tested in the hermaphrodite mating frequency assay.

***glb-5* transgenic rescue experiment with HW allele in N2 background**

Because *glb-5* is located in the *mate-1* QTL interval and recent studies have identified *glb-5* polymorphisms that regulate *C. elegans* behavior (Persson *et al*. 2009, McGrath *et al.* 2009), we tested whether *glb-5* was the causal gene underlying *mate-1*. Whereas the Bristol allele of *glb-5* contains a putative null mutation, the HW *glb-5* locus appears to encode a functional allele. We measured mating frequency of transgenic lines where *glb-5(HW)* is ectopically expressed under its native promoter in an N2 background. Because these transgenic lines also carried a HW introgression around *npr-1* (CX10872 and CX10873) (McGrath *et al.* 2009), we first backcrossed the lines to N2 for two generations to replace *npr-1*(*HW*) with *npr-1*(*Bri)*, while maintaining the *glb-5(HW)* transgene. The resulting backcross lines (ZC1902 and ZC1903) did not exhibit aggregation behavior and were genotypically confirmed to be homozygous for *npr-1(Bri)* on the X chromosome. Further, neuronal GFP expression (McGrath *et al.* 2009) in our backcross lines confirmed the presence and appropriate expression of the *glb-5* transgene. We observed that there was no increase in hermaphrodite mating frequency in the two lines carrying the *glb-5(HW)* transgene, in comparison with the non-transgenic siblings or the N2 control (**Figure S6**). Because this transgene was shown to be sufficient to rescue oxygen and carbon dioxide-regulated locomotory behaviors in the previous study (McGrath *et al.* 2009), our results suggest that *glb-5* is not the gene underlying the *mate-1* QTL.

Supplementary References

Broman, K. W. and S. Sen 2009 *A Guide to QTL Mapping with R/qtl*. Springer, New York.

Persson, A., *et al*. 2009 Natural variation in a neural globin tunes oxygen sensing in wild *Caenorhabditis elegans*. Nature 458: 1030-1033.
