# Supplementary material for: When Females Produce Sperm: Genetics of C. elegans Hermaphrodite Reproductive Choice
Source: G3 (Bethesda). 2013 Oct 1;3(10):1851–9. doi: 10.1534/g3.113.007914 (PMC3789810; doi:10.1534/g3.113.007914)
Supplement: Supporting Information [file supp_g3.113.007914_FigureS1.pdf]

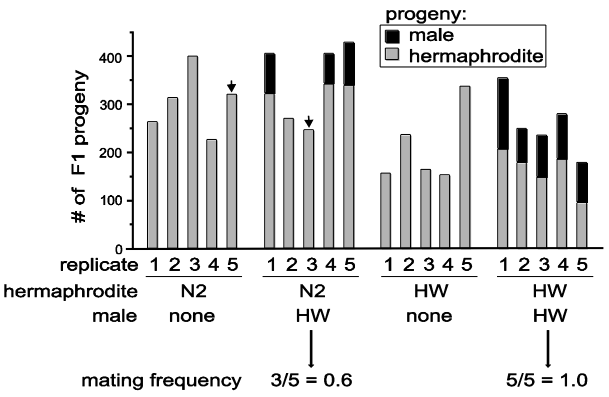

Figure S1. Quantification of hermaphrodite and male progeny in mating frequency assay
A control experiment depicting the outcome of alternative hermaphrodite reproductive strategies. In each replicate, one L4 hermaphrodite is placed alone or paired with one L4 male in an arena. During the test period, the hermaphrodite may mate with the male, resulting in a large number of male progeny ('mating success'), or only self-reproduce, resulting in nearly 100% hermaphrodite progeny ('mating failure'). Mating frequency is defined as the proportion of tested pairs bearing large numbers of male F1 progeny. Hermaphrodites tested alone, where self-reproduction is the only option, produce zero or, occasionally, very few males due to X-chromosome nondysjunction. Arrowheads depict the two replicates where a single F1 male was observed among progeny, showing that the effective male progeny rate from nondysjunction is orders of magnitude less than that from mating in our assay.
