# Supplementary material for: When Females Produce Sperm: Genetics of C. elegans Hermaphrodite Reproductive Choice
Source: G3 (Bethesda). 2013 Oct 1;3(10):1851–9. doi: 10.1534/g3.113.007914 (PMC3789810; doi:10.1534/g3.113.007914)
Supplement: Supporting Information [file supp_g3.113.007914_FigureS2.pdf]

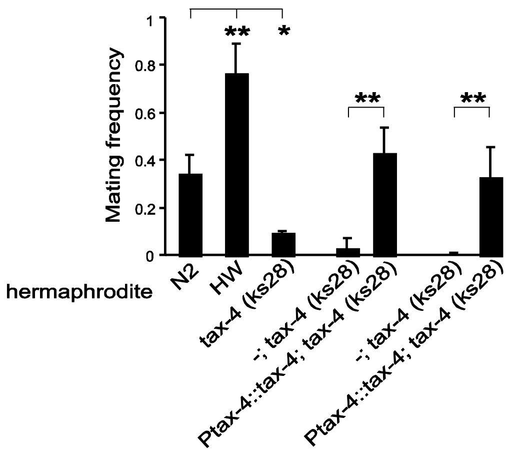

Figure S2. Transgenic rescue of the *tax-4 (ks28)* mutant recapitulates wild-type N2 hermaphrodite mating frequency

Mating frequency of two transgenic strains, ZC2302 and ZC2303 [and their nontransgenic siblings, depicted by a 'minus' sign], expressing a full-length N2 *tax-4* genomic DNA fragment in a *tax-4 (ks28)* background. Bar graphs depict mean±SEM of multiple trials. *$p<0.05$ and **$p<0.01$ by permutation test stratified by trial.
