# Supplementary material for: When Females Produce Sperm: Genetics of C. elegans Hermaphrodite Reproductive Choice
Source: G3 (Bethesda). 2013 Oct 1;3(10):1851–9. doi: 10.1534/g3.113.007914 (PMC3789810; doi:10.1534/g3.113.007914)
Supplement: Supporting Information [file supp_g3.113.007914_FigureS3.pdf]

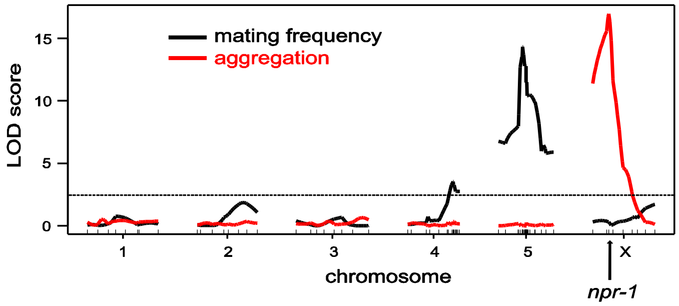

Figure S3. QTL mapping of aggregation behavior and hermaphrodite mating frequency variation between N2 and HW

As a control, variation in aggregation behavior between N2 and HW was mapped to a marker adjacent to *npr-1*. Using the same RILs, mating frequency mapped to two QTL, *mate-1* and *mate-2*, on chromosomes V and IV, respectively, but did not map to *npr-1*. In this analysis, aggregation was scored and mapped as a binary trait. Dotted line depicts significance threshold determined by permutation of the data.
