# Supplementary material for: When Females Produce Sperm: Genetics of C. elegans Hermaphrodite Reproductive Choice
Source: G3 (Bethesda). 2013 Oct 1;3(10):1851–9. doi: 10.1534/g3.113.007914 (PMC3789810; doi:10.1534/g3.113.007914)
Supplement: Supporting Information [file supp_g3.113.007914_FigureS4.pdf]

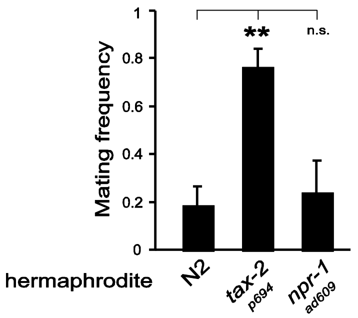

Figure S4. The *npr-1 (ad609)* mutant does not exhibit increased hermaphrodite mating frequency

The *npr-1 (ad609)* mutant, which displays bordering and aggregation behaviors, does not exhibit increased hermaphrodite mating frequency relative to N2, demonstrating that bordering and aggregation are not sufficient to cause high mating. The *tax-2 (p694)* mutant is included here as a high mating control. Bar graphs depict mean±SEM of multiple trials. **p<0.01 by permutation test stratified by trial; n.s., not-significant.
