# Supplementary material for: When Females Produce Sperm: Genetics of C. elegans Hermaphrodite Reproductive Choice
Source: G3 (Bethesda). 2013 Oct 1;3(10):1851–9. doi: 10.1534/g3.113.007914 (PMC3789810; doi:10.1534/g3.113.007914)
Supplement: Supporting Information [file supp_g3.113.007914_FigureS5.pdf]

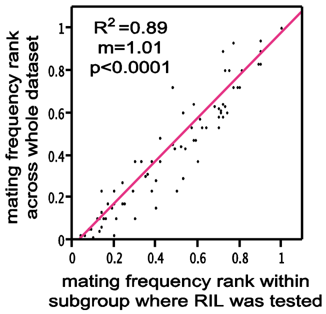

Figure S5. Relationship between rank percentile measurements of mating frequency of RILs within subgroups tested and the combined dataset

Mating frequency of 158 RILs was measured in subgroups of 22-25 RILs. Mating frequency data were used to calculate rank assignments for each RIL within subgroup tested (X-axis) and across the 158 RIL combined dataset as a whole (Y-axis). The strong linear correlation observed suggests that data collected as subgroups could be combined for QTL analysis.
