# Supplementary material for: When Females Produce Sperm: Genetics of C. elegans Hermaphrodite Reproductive Choice
Source: G3 (Bethesda). 2013 Oct 1;3(10):1851–9. doi: 10.1534/g3.113.007914 (PMC3789810; doi:10.1534/g3.113.007914)
Supplement: Supporting Information [file supp_g3.113.007914_FigureS6.pdf]

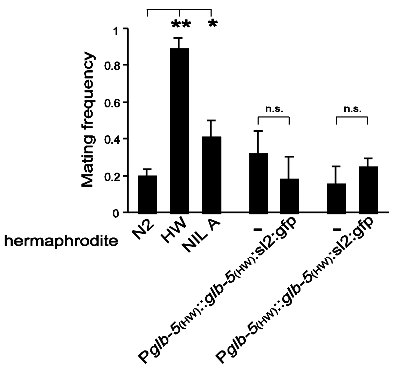

Figure S6. Transgenic line experiments do not implicate *glb-5* as the causal gene underlying the *mate-1* QTL

Mating frequency of two transgenic strains, ZC1902 and ZC1903 [and their nontransgenic siblings, depicted by a 'minus' sign], carrying ectopic expression of *glb-5(HW)* in an N2 background. These transgenic constructs conferred *glb-5*-dependent phenotypes in a previous study (McGrath *et al*. 2009), but had no effect on mating frequency here. Bar graphs depict mean±SEM of multiple trials. *p<0.05 and **p<0.01 by permutation test stratified by trial; n.s., not significant.
